# Effects of a physical activity program from diagnosis on cardiorespiratory fitness in children with cancer: a national non-randomized controlled trial

**DOI:** 10.1186/s12916-020-01634-6

**Published:** 2020-07-06

**Authors:** Martin Kaj Fridh Nielsen, Jesper Frank Christensen, Thomas Leth Frandsen, Troels Thorsteinsson, Lars Bo Andersen, Karl Bang Christensen, Peder Skov Wehner, Henrik Hasle, Lis Ørgaard Adamsen, Kjeld Schmiegelow, Hanne Bækgaard Larsen

**Affiliations:** 1 Department of Pediatrics and Adolescent Medicine, The Juliane Marie Center, University Hospital of Copenhagen (Rigshospitalet), Blegdamsvej 9, DK-2100 Copenhagen, Denmark; 2 The University of Copenhagen, Faculty of Health Science, Institute for Clinical Medicine, Copenhagen, Denmark; 3 Center for Inflammation and Metabolism/Center for Physical Activity (CIM/CFAS), University Hospital (Rigshospitalet), Copenhagen, Denmark; 4 Department of Sports Medicine, Norwegian School for Sports Sciences, Oslo, Norway; 5 Faculty of Education, Arts and Sports, Department of Sport, Food and Natural Sciences,Western Norway University of Applied Sciences, Post box 133, 6851 Sognal, Norway; 6 Department of Biostatistics, University of Copenhagen, Copenhagen, Denmark; 7 Department of Pediatric Hematology and Oncology, H.C. Andersen Children’s Hospital, Odense University Hospital, Odense, Denmark; 8 Pediatrics and Adolescent Medicine, Aarhus University Hospital, Aarhus, Denmark; 9 Faculty of Health Science, Department of Public Health, Institute for Clinical Medicine, The University of Copenhagen, Copenhagen, Denmark; 10 The University Hospitals Centre for Health Research (UCSF), University Hospital (Rigshospitalet), Copenhagen, Denmark

**Keywords:** Childhood cancer, Exercise, Cardiorespiratory fitness

## Abstract

**Background:**

Children with cancer experience impaired cardiorespiratory fitness and physical function during and after treatment restricting their possibilities to engage in social activities including sport, leisure activities, and school. The objectives were to determine the effects of classmate-supported, controlled, supervised, in-hospital, physical activity program to preserve cardiorespiratory fitness and physical function from time of diagnosis in children with cancer.

**Methods:**

National non-randomized controlled trial including schoolchildren aged 6–18 years at diagnosis treated with chemo-/radiotherapy. We included 120 of 128 eligible patients (94%) in the intervention group (62.5% boys, 11.2 ± 3.1 years) from East Denmark and 58 patients in the control group (57% boys, 11.0 ± 3.2 years) from West Denmark. Eight children from the control group withdrew from participation. The groups were comparable in anthropometrics and cancer diagnoses (*p* > 0.05). The intervention consisted of (i) supervised in-hospital physical activity from diagnosis and throughout intensive treatment, (ii) 90-min general educational session on cancer and therapy in the child’s school class, and (iii) selection of two classmates as ambassadors who took turns to support the child’s physical training during the daytime. The primary outcome was cardiorespiratory fitness (VO_2_peak, mL/min/kg) at 6 months after diagnosis (sex, age, diagnosis adjusted). Secondary outcomes were sit-to-stand, timed-up-and-go, handgrip strength, and balance test scores.

**Results:**

Ambassadors were identified for all, and 2542 individual and 621 group training sessions were held. VO_2_peak deteriorated over time in the control group (− 0.17 [95% CI − 0.32 to − 0.02] per week, *p* = 0.02), but not in the intervention group (*p* = 0.14). At 6 months from diagnosis, VO_2_peak was higher in the intervention group (29.6 ± 5.6 mL/kg/min) than in the control group (22.1 ± 5.6 mL/kg/min) (*p* = 0.01), and the intervention group had a better physical function at 3 and 6 months (*p* < 0.0001).

**Conclusions:**

Peer-supported, supervised, in-hospital, physical activity is safe and feasible in children with cancer during treatment. Further, the results suggest that the intervention might mitigate impairments in cardiorespiratory fitness during treatment in children with cancer.

**Trial registration:**

The study was prospectively registered on the 11 January 2013. Clinicaltrial.gov NCT01772849 and NCT01772862.

## Background

As childhood cancer survival rates continue to improve, there is a growing need to reduce treatment-related complications [1, 2], including social, academic, and physical functioning [3]. A prevalent and serious long-term consequence of childhood cancer treatment is impaired physical function with limited ability to perform daily tasks, impaired self-perception [4, 5], and reduced capacity to fully participate in social activities, including school [6, 7]. These disabilities are associated with poor health [8] and increased mortality [9].

The negative impact of childhood cancer and its treatment includes impaired cardiorespiratory fitness [10–12], muscle strength [13–16], and balance [17], along with prolonged absence from school, sport, and leisure activities, thus dramatically reducing peer interactions [18, 19]. As the development of social skills and social bonds with peers is disrupted [18, 19], the children become vulnerable to social exclusion [20], further decreasing their incentive to be physically active [21, 22]. This results in reduced health-related quality of life [14, 23]. Accordingly, there is an unmet need for preemptive interventions that strengthen all these aspects of the child’s life. We designed a multicomponent intervention that included a supervised in-hospital physical activity program combined with co-admissions of healthy classmates as *ambassadors* to support the children with cancer [24] and promote social network links between hospital, school, and peers. The intervention was initiated at diagnosis to preserve preexisting relationships and physical function as ambassadors can increase motivation for engaging in exercise-professional supervised physical activity [25–27].

## Methods

### Objectives

- The primary objective of this study was to investigate the effects of the intervention on cardiorespiratory fitness and physical function after 6 months of treatment between children with cancer in the intervention group and children with cancer in the control group.
- The secondary objectives of this study were (i) to compare children with cancer’s cardiorespiratory fitness with a historic healthy age- and sex-matched control group, and (ii) to compare children with cancer’s physical function with a subgroup of healthy age- and sex-matched classmates.

### Design and setting

This study is part of the ongoing “Rehabilitation including Social and Physical Activity and Education in Children and Teenagers with Cancer” (RESPECT) study (Clinical Trial registration NCT01772849 and NCT01772862), a nationwide, prospective, non-randomized controlled multicomponent study. The study is embedded in the work structure of the Center for Integrated Rehabilitation [28]. The RESPECT study was initiated to simultaneously address the level of education, social function, cardiorespiratory fitness, and physical function in children with cancer [24].

### Participants

**Fig. 1:**
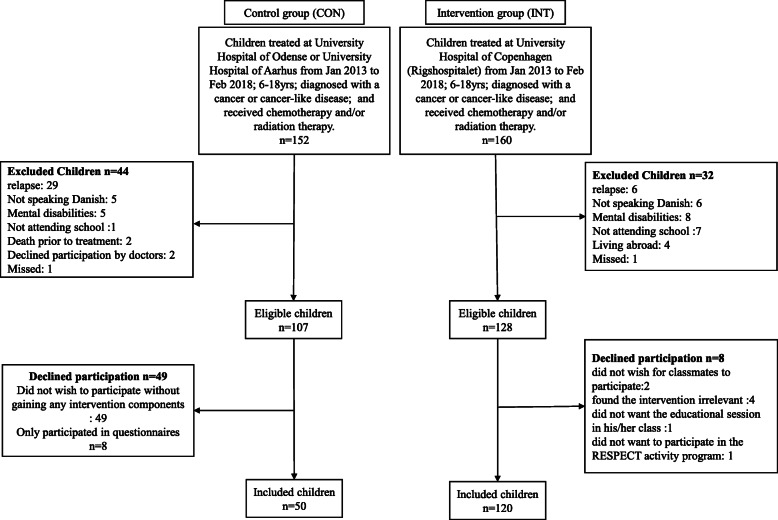
CONSORT diagram of the enrollment process in the RESPECT study. RESPECT, Rehabilitation including Social and Physical Activity and Education in Children and Teenagers with Cancer

As presented in Fig. 1, we consecutively included participants between January 2013 and February 2018. Inclusion criteria were 6–18 years; any cancer diagnosis or Langerhans cell histiocytosis (LCH) or myelodysplastic syndrome (MDS); treated with chemotherapy and/or radiation therapy; enrolled in school at diagnosis; and able to communicate in Danish. Exclusion criteria were mental disability (e.g., Down syndrome) and severe co-morbidity. Children treated at the University Hospital of Copenhagen were included in the intervention group. Children treated at Odense University Hospital and Aarhus University Hospital were included in the control group and received standard institutional guided care.

### Intervention components

The intervention consisted of three components. Firstly, a 90-min educational session was held in the child with cancer class on cancer etiology, the treatment, and its side effects; supportive care; everyday life at the hospital; the importance of physical activity; and the RESPECT study. Secondly, two classmates were selected as “ambassadors” in collaboration with the teachers, the classmates’ parents, and the child with cancer [29]. The motivation for becoming ambassadors primarily consists of pre-existing friendships and wanting to help a classmate in need (the Good Samaritan) [29]. The ambassadors were invited to be co-admitted every 14th in- and out-patient day throughout the entire treatment period. The ambassadors were co-admitted to the hospital for the day (i.e., 9 a.m. to 3 p.m.) and were present during the daily routines at the ward and participated in school, social, and physical activities. The main role of the ambassadors was to provide peer-support and increase the child with cancer’s motivation to engage in school and physical activities. In Additional file 1, we present a flow chart of how an ambassador co-admission was planned. Thirdly, an in-hospital supervised physical activity intervention (the RESPECT physical activity program) was run during admission to the Department of Pediatric Oncology.

**Fig. 2:**
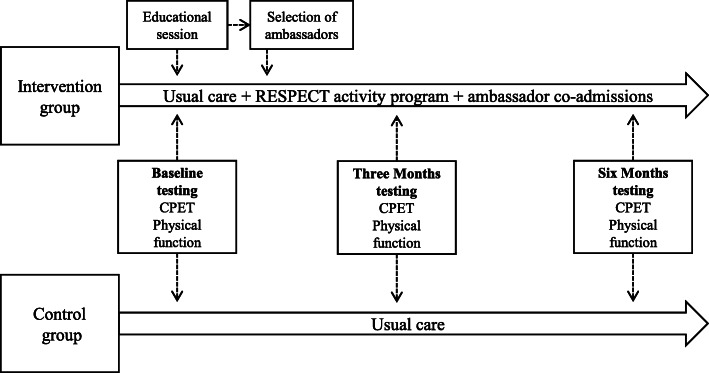
Flow chart of the study timeline

The RESPECT physical activity program consisted of individually designed activities (duration 5–30 min) offered thrice a week (Monday, Wednesday, and Friday) and group sessions (duration 30–120 min) including all eligible children with cancer and their ambassadors on Tuesdays and Thursdays (Table 1). The ambassadors were included to increase the motivation of the child with cancer. Each session was designed to accommodate the current well-being (e.g., nausea, pain, dizziness) and physical capacity of the child with cancer. Each session started with cardiorespiratory fitness exercises spanning from simple mobilization to targeted aerobic exercises (if the child’s well-being permitted) followed by exercises and/or games designed to improve muscle strength and balance [30]. Additional file 2 outlines the exercises from which games were developed. Key equipment consisted of stationary cycle-ergometers, treadmills, dumbbells, balls, and various other pieces to create games. The intensity during group sessions has been reported elsewhere [10]. The mean heart rate was 145 beats/min [95% CI 142 to 149] or 69.3% [68.1 to 70.4%] of age-specific predicted maximal heart rate. The maximal heart rate was 185 beats/min [95% CI 174 to 184] or 89% [95% CI 87.7 to 90.4%] of age-specific predicted maximal heart rate [10]. In Fig. 2, we present the flow of the study.

**Table 1:** The in-hospital RESPECT activity program

Training/weekday	Monday	Tuesday	Wednesday	Thursday	Friday	Weekends
Able to walk/not isolated	Individual session 5–30 min Cardiorespiratory fitness Muscle strength Balance	Group session 30–120 min Cardiorespiratory fitness Muscle strength Balance	Individual session 5–30 min Cardiorespiratory fitness Muscle strength Balance	Group session 30–120 min Cardiorespiratory fitness Muscle strength Balance	Individual session 5–30 min Cardiorespiratory fitness Muscle strength Balance	No training
Able to walk/isolated	Individual session 5–30 min Cardiorespiratory fitness Muscle strength Balance	Individual session 5–30 min Cardiorespiratory fitness Muscle strength Balance	Individual session 5–30 min Cardiorespiratory fitness Muscle strength Balance	Individual session 5–30 min Cardiorespiratory fitness Muscle strength Balance	Individual session 5–30 min Cardiorespiratory fitness Muscle strength Balance	No training
Bedridden	Individual session 5–30 min Muscle strength	Individual session 5–30 min Muscle strength	Individual session 5–30 min Muscle strength	Individual session 5–30 min Muscle strength	Individual session 5–30 min Muscle strength	No training

*RESPECT* Rehabilitation including Social and Physical Activity and Education in Children and Teenagers with Cancer

### Healthy age- and sex-matched control children

Between August 2017 and February 2018, we consecutively included all ambassadors in an age- and sex-matched healthy control group to investigate the differences in physical function (sit-to-stand, timed-up-and-go, flamingo balance, and handgrip strength) between children with cancer and healthy children. Further, we randomly paired each child with cancer who completed a cardiopulmonary exercise test (CPET) with five age- and sex-matched children (*n* = 255) from the European Heart Study and The Copenhagen School Child Intervention Study [31–35]. These studies include children from Denmark, Norway, Estonia, and Portugal [31–35] and consist of 9642 children aged 6–18 years who have all completed CPET.

### Anthropometry, body composition, and medical characteristics

Table 2 shows the anthropometric and clinical characteristics of the included children. We categorized the children’s cancers as oncological diseases (extracranial solid tumors and tumors located in the central nervous system) and hematological diseases (leukemia, lymphoma, LCH, and MDS).

**Table 2:** Anthropometric and clinical characteristics

Anthropometric characteristics	Intervention (*n* = 120)	Control (*n* = 50)	*p* value	Ambassadors (*n* = 62)	Healthy age- and sex-matched children (*n* = 255)
Sex (males/females)	75/45 (62.5%/37.5%)	31/23 (57%/43%)	0.61	37/25 (60%/40%)	180/75 (71%/29%)
Age (years)	11.2 ± 3.1	11.0 ± 3.2	0.44	11.9 ± 2.5	12.9 ± 3.1
Height (m)	1.52 ± 0.19	1.50 ± 0.2	0.69	1.57 ± 0.16	1.58 ± 0.19
Weight (kg)	44.8 ± 17.2	41.0 ± 14.0	0.21	48.4 ± 16.2	49.4 ± 16.9
BMI (kg/m^2^)	18.5 ± 4.0	17.6 ± 2.5	0.10	19.0 ± 3.6	17.9 ± 5.2
**Diagnosis**					
Leukemia	47 (39%)	23 (46%)			
Lymphoma	22 (18%)	8 (16%)			
Extracranial solid tumors	35 (29%)	14 (28%)			
Central nervous system tumor	11 (9%)	5 (10%)			
Other hematological disease	5 (4%)	0 (0%)	0.61		
**Treatment protocols**					
NOPHO ALL 2008	34	20			
NOPHO-DBH-AML 2012	11	3			
ICC APL 01	2	0			
Euro-LB-02	4	0			
Euro NET PHL-C1 interrim	6	0			
Euro NET PHL-C2	2	4			
BFM NHL 2004	7	0			
BFM NHL 2013	3	4			
Euro-Ewing 99	11	2			
EURAMOS-1	6	4			
CCLG interim	1	0			
EpSSG RMS 2005	7	4			
EpSSG-NRSTS 2005	5	0			
UKSSG	0	1			
SIOPEL 6- SR	1	0			
SIOPEL. high risk-PLADO	1	0			
Neoadjuvant (docetaxel/cisplatin/fluorouracil)	1	0			
SIOP-CNS GCT 2	3	2			
SIOP ependynoma 2	2	0			
(EU-RHAB) 2009	1	1			
Angiocomb	0	1			
Herby study	0	2			
SIOP PNET 5	3	1			
SIOP- LGG 2004	1	1			
LCH-III	1	0			
Allogeneic transplantation	4	0			
Other	1	0			
No chemotherapy	2	0			
**Treatment modalities**					
Chemotherapy	118	50			
Radiation therapy	25	10			
Surgery	40	18			
**Tumor location**					
Central nervous system	11	5			
Head	6	3			
Torso	13	6			
Upper extremity	3	0			
Lower extremity	13	5			

Descriptive data are presented as mean and standard deviations or frequency and percentage

### Physical tests

The primary outcome was VO_2_peak measured with the cardiopulmonary exercise test (CPET) and the secondary outcomes were timed-up-and-go, sit-to-stand, flamingo balance, and handgrip strength. The tests were carried out within 31 days of diagnosis (baseline), 3 months after diagnosis ± 30 days, and 6 months after diagnosis ± 30 days. The treating physician permitted the tests providing the child’s thrombocyte count was > 10 billion/L, hemoglobin count was > 5 mmol/L, and the temperature was < 38 °. Exclusion criteria (for testing) included active diarrhea, having a cough or a cold, and side effects preventing testing. Annual meetings were held with all centers to ensure comparability, and instruction videos were distributed to all members of the test teams. The tests are described in detail elsewhere [24].

*CPET* was performed on an electronically braked cycle ergometer (Lode Corival Pediatric or Monark Ergomedic 839 E) following a modified Godfrey protocol [24, 36]. Ventilation and gas exchange data were determined breath-by-breath (INNOCOR ergo-spirometry-system, INNO00010, Innovision, DK-5260 Odense, Denmark or Jaeger Master Screen® CPX System (MS-CPX), and JLAB Software Package™). VO_2_peak was defined as the highest mean over 60 s and expressed in milliliters/kilogram/minute (ml/kg/min). Maximal watts of the test was recorded. Heart rate and oxygen saturation were measured every 30 s (Polar FT2 sport tester Polar Electro, Kemple, Finland). One subjective criterion and two objective criteria were required for the CPET test to be considered valid. The subjective criteria were signs of intense effort. The objective criteria were heart rate > 180 beats/min, and respiratory exchange ratio > 1.05. The test was stopped if oxygen saturation was under 90 or the child was unable to maintain the minimum required tempo (70 rpm). We compared the results with data collected in the European Heart Study and The Copenhagen School Child Intervention Study include healthy children from Denmark, Norway, Estonia, and Portugal [31–35]. The European Heart Study used a similar CPET protocol as this study, and The Copenhagen School Child Intervention Study used a progressive treadmill running test, and the results were adjusted to account for the disparity between a bicycle and a running test [37].

### Physical function tests

The children performed the timed-up-and-go 3-m test [38] three times and the last score was analyzed. The children performed the sit-to-stand [39] and the score equated the number of repetitions after 30 s. The children performed the flamingo balance [40] barefooted and on one leg (preferred) for 60 s. The number of restarts was recorded.

*Handgrip strength* was performed twice per arm and the highest score was analyzed (Saehan hand dynamometer, Glanford Electronics, Scunthorpe, UK) [41]. The results are expressed in kilograms.

### Ethics approval and consent to participate

All participants and their parents received oral and written information and the parents gave written informed consent to participate in the educational sessions, the inclusion of ambassadors, and the participation in the RESPECT activity program. The Regional Ethics Committee for the Capital Region (file. H 3-2012-105) and the Danish Data Protection Agency (file. 2007-58-0015/nr.30-0734) approved the study and the data protection structure.

### Statistics

The power calculation is based on the primary endpoint 1 year after ended treatment being VO_2_peak and the power calculation is based on an anticipated 10% higher VO_2_peak in the intervention group compared to the control group 1 year after cessation of treatment. We based the power calculation on a pilot that found a baseline VO_2_peak of 24.3 (SD 5.9) [42]. The significance level 1 year after the treatment ended was 0.025 and the power was 0.90 resulting in 120 children in each group [24]. All analyses were performed in R (version 3.6.0) and R-studio. VO_2_peak, watt max, timed-up-and-go, sit-to-stand, and handgrip strength were analyzed using a linear mixed model to evaluate differences in change over time between the intervention and the control group. Flamingo balance was analyzed using random-effects Poisson regression. Fixed effects were age, sex, and cancer disease (solid tumors versus hematological cancers), and the random effect was participant ID. We performed post hoc comparisons using the Bonferroni test, adjusting the *p* value by the number of comparisons (*p* = 0.05/3 = 0.017). One-way ANOVA was used to analyze differences at baseline between patient groups (intervention and control) vs. the healthy age-and sex-matched group and used the Bonferroni test adjusting the *p* value accordingly (*p* = 0.05/3 = 0.017).

The ambassadors represented an age- and sex-matched group regarding physical function. Each child who completed a CPET was age- and sex-matched with five healthy children recruited in previously published studies [31–35]. Differences in the proximity to diagnosis the children were tested were analyzed using unpaired *t* test. Correlations between time (days from diagnosis to baseline testing) and physical function parameters were analyzed using Spearman’s rank correlation. Training frequency was calculated by dividing the number of days with physical activity by the number of weekdays admitted to the department of pediatric oncology (excluding weekends and holidays). Anthropometric data and results from the statistical analysis were presented as mean and standard deviations or median and interquartile range as best applied. The significance level was *p* < 0.05.

## Results

### Baseline characteristics

We included 120 of 128 (94%) eligible children in the intervention group and 58 of 107 (54%) eligible children in the control group. No child withdrew from the intervention group, while eight children withdrew from the control group. The reasons for declining participation are presented in Fig. 1. No difference was observed in age, sex, height, weight, BMI, or diagnosis distribution between groups. Anthropometric and clinical characteristics are presented in Table 2.

### Ambassadors

We included 246 ambassadors (41% girls) and at least one ambassador was identified for all children in the intervention group (median 2, range 1 to 4). Of the 246 ambassadors, 63 of the latest appointed ambassadors were included in a subgroup that performed the physical function tests. No ambassador withdrew from the study during the first 6 months of treatment. The ambassador’s supportive impact on the child with cancer has previously been reported in detail and provides the child with cancer an opportunity to engage in friendly competition and the possibility to receive peer-support. Besides the support from parents and an exercise professional, the children stated that the ambassadors were the most important motivational factor for engaging in physical activities during treatment [43].

### Training frequency, safety, and feasibility

In total, 2542 individual training sessions and 621 group training sessions were held. The median number of attended sessions per child was 23 [range 4 to 84] training sessions corresponding to a participation rate of median 68% [11% to 100%] or three sessions per 5 days of in-hospital admissions (excluding weekends and holidays). Six minor events occurred during the intervention: four children experienced minor bruising, one child had a nosebleed during warm-up, and one child fainted shortly after exercise but had no further complications. The feasibility of performing the CPET was low and 138 of 510 (27.1%) possible tests were completed. We divided the reasons for not completing the tests into six categories: (1) Not safe to perform the test—Before each test, we consulted the treating physician regarding the safety of performing the tests and we followed the treating physician’s recommendations on all tests; (2) Unable to perform the test—Before each test, we evaluated whether the child would be able to perform the tests, based on the child’s general well-being (i.e., the presence of treatment-related side effects such as nausea, pain, and dizziness) and physical capacity (i.e., the ability to stand and walk); (3) Not motivated—When the child had no apparent physical reason not to perform the test; (4) Logistical reasons—When the child received concurrent medical procedures that prevented testing or received treatment abroad. Additionally, the CPET was not available at Odense University Hospital; (5) Equipment issues—When the equipment failed during a test or during equipment maintenance periods; and (6) Late inclusion—When a child was included at a late time point in their treatment. The two main reasons for not performing the CPET were not safe to perform the test (15.9% of all possible tests) and unable to perform the tests (35.3% of all possible tests). A detailed description regarding reasons not to perform all tests at each time point is presented in Additional file 3.

### Effect of the RESPECT activity program

We observed a significant difference in changes over time in VO_2_peak (ml/min/kg) in favor of the intervention group (0.25 [95% 0.07 to 0.43] ml/min/kg per week, *p* = 0.006) (Table 3). The intervention group performed significantly better 6 months after diagnosis compared with the control group (29.6 ± 6.9 vs 22.1 ± 5.8 ml/min/kg, *p* = 0.0146, Table 3). VO_2_peak tended to diminish over time in the control group (− 0.19 [95% CI − 0.35 to − 0.03] ml/min/kg per week, *p* = 0.018), whereas this was not the case in the intervention group (0.06 [95% CI − 0.02 to 0.15] ml/min/kg per week, *p* = 0.14) (Additional file 4). We observed a significant difference in changes over time in watt max (watts) in favor of the intervention group (0.21 [95% CI 0.07 to 0.35] max watt per week, *p* = 0.0038). However, there was no difference between groups at 3 months or 6 months (Table 3). We found no difference in changes over time in timed-up-and-go, sit-to-stand, or handgrip strength. However, the intervention group performed better at all time points (Additional file 4).

**Table 3:** Effects of the RESPECT activity program on cardiorespiratory fitness and physical function

	*n*	Baseline	*n*	3 months	*n*	6 months	Group (*p*)	Time (*p*)	Change over time between groups per week (mean, 95% CI)	Change over time between groups (*p*)
**VO**_**2**_**peak (mL/kg/min)**										
INT	38	27.4 ± 6.97	30	26.1 ± 6.1	34	29.6 ± 6.9	0.2830	0.1415	0.25 [0.07 to 0.43]	0.0062
CON	13	27.8 ± 8.3	13	23.8 ± 5.9	10	22.1 ± 5.8		0.0238		
**Max watt (W)**										
INT	38	107.6 ± 33.1	30	95.5 ± 34.9	30	112.6 ± 49.5	0.0438	0.0185	0.21 [0.07 to 0.35]	0.0038
CON	13	113.1 ± 63.8	13	90 ± 45.1	10	69 ± 27		0.0694		
**Sit-to-stand (reps)**										
INT	90	25.1 ± 6.4	74	24.2 ± 7.2	81	24.6 ± 8.1	< 0.0001	0.4960	0.04 [− 0.08 to 017]	0.5057
CON	26	18.0 ± 5.0	38	16.5 ± 6.8	28	17.6 ± 7.3		0.8919		
**Timed-up-and-go** (s)										
INT	85	4.0 ± 0.8	74	4.3 ± 1.1	82	4.1 ± 1.2	< 0.0001	0.3038	0.02 [− 0.09 to 0.04]*	0.4129
CON	27	5.5 ± 1.7	38	6.0 ± 1.9	27	6.4 ± 3.3				
**Right handgrip strength (kg**)										
INT	103	21.4 ± 11.4	90	19.3 ± 10.5	91	20.1 ± 11.4	0.3418	0.4085	− 0.01 [− 029 to 0.27]	0.9438
CON	28	17.1 ± 9.7	39	12.1 ± 8.2	30	12.7 ± 8.5		0.0711		
**Left handgrip strength (kg)**										
INT	104	20.0 ± 10.8	90	17.4 ± 10.2	90	18.5 ± 10.7	0.0032	0.0062	0.01 [−0.002 to 0.02]	0.1668
CON	28	16.6 ± 10.4	39	10.5 ± 7.6	30	12.0 ± 8.4		0.0466		
**Flamingo balance (hits)**										
INT	98	0 [0 to 7]	86	0 [0 to 18]	87	0 [0 to 16]	< 0.0001	< 0.0001		0.5846
CON	27	0 [0 to 13]	37	1 [0 to 26]	27	1 [0 to 26]		< 0.0001		

VO_2_peak, sit-to-stand, timed-up-and-go, and handgrip strength are reported in mean and standard deviation, and flamingo balance is reported in median and range. All analysis is adjusted for age, sex, and diagnosis (hematological versus oncological)

*INT* the intervention group, *CON* the control group

*The interaction effect in TUG is presented in percentages

### Timing of tests and association with physical function

There was no correlation between days from diagnosis until the first test was performed concerning VO_2_peak (*p* = 0.5), max watt (*p* = 0.5), or handgrip strength (*p* = 0.9). Thus, although the intervention group was tested at a median of 12 days from diagnosis and the control group at a median of 27 days after diagnosis, this is unlikely to have influenced the results of these tests. However, there was a significant correlation between days from diagnosis and sit-to-stand (*r* = − 0.31, *p* < 0.001) and timed-up-and-go (*r* = 0.44, *p* < 0.001).

### Cardiorespiratory fitness and physical function compared with age- and sex-matched healthy control children

At baseline, both the intervention and control group performed significantly worse than the healthy age- and sex-matched children in CPET, sit-to-stand, timed-up-and-go, and handgrip strength (Table 4).

**Table 4:** Comparisons of cardiorespiratory fitness and physical function between children with cancer and healthy age-and sex-matched children at baseline (median of 12 days in the intervention group and 27 days in the control group)

	*n*	Intervention group	*p* value	*n*	Control group	*p* value	*n*	Subgroup of ambassadors	*n*	Historic healthy age- and sex-matched children
**VO**_**2**_**peak(ml/min/kg)**	38	27.4 ± 6.97	< 0.0001	13	27.8 ± 8.30	< 0.0001			255	47.7 ± 7.7
**Sit-to-stand repetitions)**	90	25.1 ± 6.4	< 0.0001	26	18.0 ± 5.0	< 0.0001	62	31.8 ± 4.5		
**Timed-up-and-go (seconds)**	85	4.0 ± 0.8	< 0.0001	27	5.5 ± 1.7	< 0.0001	61	3.3 ± 0.4		
**Right handgrip strength (kg)**	103	21.4 ± 11.4	0.002	28	17.1 ± 9.7	< 0.0001	63	26.8 ± 12.8		
**Left handgrip strength (kg)**	104	20.0 ± 10.8	< 0.0001	28	16.6 ± 10.4	< 0.0001	63	24.3 ± 11.8		

VO_2_peak, sit-to-stand, timed-up-and-go, and handgrip strength are reported in mean and standard deviation. The *p* values represent a comparison between a patient group (the intervention group and the control group) and a group of healthy children (the subgroup of ambassadors or historic healthy age- and sex-matched children)

## Discussion

In this nationwide, prospective non-randomized controlled multicomponent study, we show for the first time that a supervised, peer-supported, in-hospital physical activity program is feasible already from the time of diagnosis and that it might mitigate the impairments in cardiorespiratory fitness experienced by children with cancer, which provides a basis for a more normal everyday life during treatment. Further, the higher cardiorespiratory fitness and physical function after 6 months suggest that the children may require less rehabilitation after treatment ends to regain normal cardiorespiratory fitness and physical function. Ultimately, for childhood cancer survivors, this may improve the re-entry into everyday life, including physical activities, social interactions, school attendance, and learning abilities. The effects of the intervention might reduce the children’s risk of developing cardiorespiratory fitness-related medical conditions years after their treatment has ended. This is supported by a recent study that showed that exercise during treatment maintained left ventricular function compared with a non-exercising control group after ended treatment [44]. A key challenge in designing physical activity programs for children with cancer is to accommodate the varying and common treatment-related side effects (e.g., nausea, pain, and dizziness). Maintaining the children’s motivation is, therefore, paramount. This is the first study to include healthy classmates as ambassadors during cancer treatment and to include them in a physical activity program. The children with cancer described how their motivation for study acceptance and physical activity had increased because their ambassadors participated in the sessions. The ambassadors provided an opportunity to engage in games and provided an opportunity to receive support from friends [43]. Including the ambassadors represents a unique opportunity to both incorporate the child’s everyday life into the hospital setting and to simultaneously increase the child’s willingness to participate in rehabilitation offers [43]. Including healthy classmates as ambassadors may be more challenging in other settings and it is therefore important to investigate different approaches to including healthy children in the physical activity program.

The disproportionate participation rates (intervention group 94%; control group 47%) reflect that most of the families in the control group who declined participation did so because of the added burden without any rewards (i.e., no ambassador visits or physical activity program). Importantly, the high participation rate in the intervention group compared with other studies that generally report participation rates of 51–66% [45, 46] most likely reflects the involvement of ambassadors, group sessions, and supervised in-hospital physical activity.

Few controlled studies have initiated a physical activity/exercise intervention at diagnosis [45–47]. The previous studies have had different approaches to exercise and have used either a home-based approach [45, 47] or a supervised in-hospital approach [44, 46]. Both studies that used a home-based approach observed no effects on cardiorespiratory fitness or physical function [45, 47]. In contrast, Fiuza-Luces colleagues (*n* = 49) showed that a supervised in-hospital approach improved muscle strength in children with solid tumors [46]. Similarly, we show (*n* = 170) that a supervised in-hospital approach has positive effects on cardiorespiratory fitness and physical function. Collectively, this suggests that rehabilitation programs should be supervised by specialized exercise/physiotherapy professionals to induce positive adaptations rather than being left for the children and their families to manage.

The intervention group performed significantly better than the control group in sit-to-stand and timed-up-and-go at baseline. A probable explanation is the influence of treatment burden and duration on physical function, as the control group underwent the physical function tests significantly later than the intervention group. Consequently, the control group received higher cumulative doses of anti-cancer treatment, including physical function-impairing chemotherapy agents (i.e., glucocorticoids, anthracyclines, and vincristine), than the intervention group before performing the baseline treatment. Further, the control group may also have experienced extended periods of bed rest and sedentary behavior because of acute treatment-related side effects (i.e., nausea, dizziness, and pain). Previous studies have shown impaired physical function following prolonged periods of bed rest and sedentary behavior [48]. Thus, the disparity in cumulative anti-cancer treatments and periods of bed rest between the intervention group and the control group likely explains the difference in physical function at baseline. Further, this significant difference in physical function at baseline might explain why we did not observe any effects of the intervention over time in physical function since the initial decline observed in the control group had already occurred at the time of baseline testing. We show that children with cancer have markedly impaired cardiorespiratory fitness, muscle strength, and physical function compared with healthy age- and sex-matched children at baseline (median 12 days after diagnosis). These findings are supported by Ness and colleagues, who showed that children with ALL have 15% reduced lower extremity muscle strength and a 39% reduced walking distance 7–10 days after diagnosis [13]. These findings underline the need for interventions to be initiated at diagnosis. The feasibility of performing the CPET test was low; thus, alternative ways to assess cardiorespiratory fitness in children with cancer is warranted. Previous studies have used the 6-min walk test to assess cardiorespiratory fitness in children with cancer [45, 49]. The 6-min walk test may be a more suitable method to assess cardiorespiratory fitness because it is less strenuous and easier to carry out than the CPET, and a recent study demonstrated that the 6-min walk test was a good predictor in childhood cancer survivors [50]. However, these results need to be verified during treatment as other physical limitations independent of cardiorespiratory fitness (i.e., muscle weakness and balance impairments) may impair the children's 6-min walk test performance.

### Strengths and limitations

The strength of this study is the high inclusion rate in the intervention group with 94% of eligible children completing the intervention. The feasibility and safety of promoting physical activity in children with cancer, independent of diagnosis, are therefore generalizable. We cannot exclude any possible geographical differences between Copenhagen and the rest of the country regarding the patients, personnel responsible for testing, and within differences in standard institutional guided care; however, each institution has the same financial resources available in the treatment of the children. Another limitation of the study is the low participation rate in the control group: only 47% participated, introducing the possible sampling bias. The disproportionate difference in the participation rate between the groups reduces the generalizability of the results. It can be speculated that the children in the control group consisted of the children with the best cardiorespiratory fitness and physical function and/or an interest in exercise, consequently resulting in an underestimation of the results. Further, the study is limited by the few completed CPET. The missing data indicate that the children with the best physical capacity completed the CPET, thus, limiting the generalizability of the effects of the intervention.

## Conclusion

Children with cancer have impaired cardiorespiratory fitness and physical function at a median of 12 days after diagnosis. This study shows that an in-hospital peer supported and exercise-professional supervised  physical activity intervention initiated from diagnosis is feasible in children with cancer. Further, the study suggests that the intervention might mitigate the impairments in cardiorespiratory fitness in children with cancer.

## Supplementary information

**Additional file 1.** Flow chart of planning an ambassador co-admissions.

**Additional file 2.** Exercise Library of which games were developed around: Overview of exercises primarily used in the RESPECT activity program. The primary focus was on Cardiorespiratory fitness, muscle strength, and stability/balance.

**Additional file 3. **Reasons why tests were not completed. **Not safe to perform test** was based on the treating physician’s evaluation of the safety of performing the tests. **Unable to perform the test** was based on an assessment of the child’s general well-being and physical capacity prior to testing. **Not motivated** if no apparent physical reason for not performing the test. **Logistical reasons** include concurrent medical procedures that prevented testing and receiving treatment abroad. Furthermore, the CPET test was not available at Odense University Hospital. **Equipment issues** include equipment failure and maintenance. **Late inclusion** includes children that were enrolled later in their treatment.

**Additional file 4. **Effects of the RESPECT activity program: Results from the linear mixed model. The first mentioned covariable serves as the reference variable in the linear mixed model. *P* < 0.017 was considered significant. INT = intervention group, CON = Control group. Oncologic consists of oncological diseases (extracranial solid tumors and tumors located in central nervous system). Hematologic consists of hematological diseases (leukemia, lymphoma, Langerhans cell histiocytosis and myelodysplastic syndrome).

## Data Availability

The datasets used during the current study are available from the corresponding author on reasonable request after the last follow-up data have been collected and published.

## Abbreviations

CPET: Cardiopulmonary exercise test
LCH: Langerhans cell histiocytosis
MDS: Myelodysplastic syndrome
RESPECT: Rehabilitation including Social and Physical Activity and Education in Children and Teenagers with Cancer
