# Supplementary figures and images for: Effects of a physical activity program from diagnosis on cardiorespiratory fitness in children with cancer: a national non-randomized controlled trial
Source: BMC Med. 2020 Jul 6;18:175. doi: 10.1186/s12916-020-01634-6 (PMC7336676; doi:10.1186/s12916-020-01634-6)

Additional file 1: Flow chart of planning an ambassador co-admissions


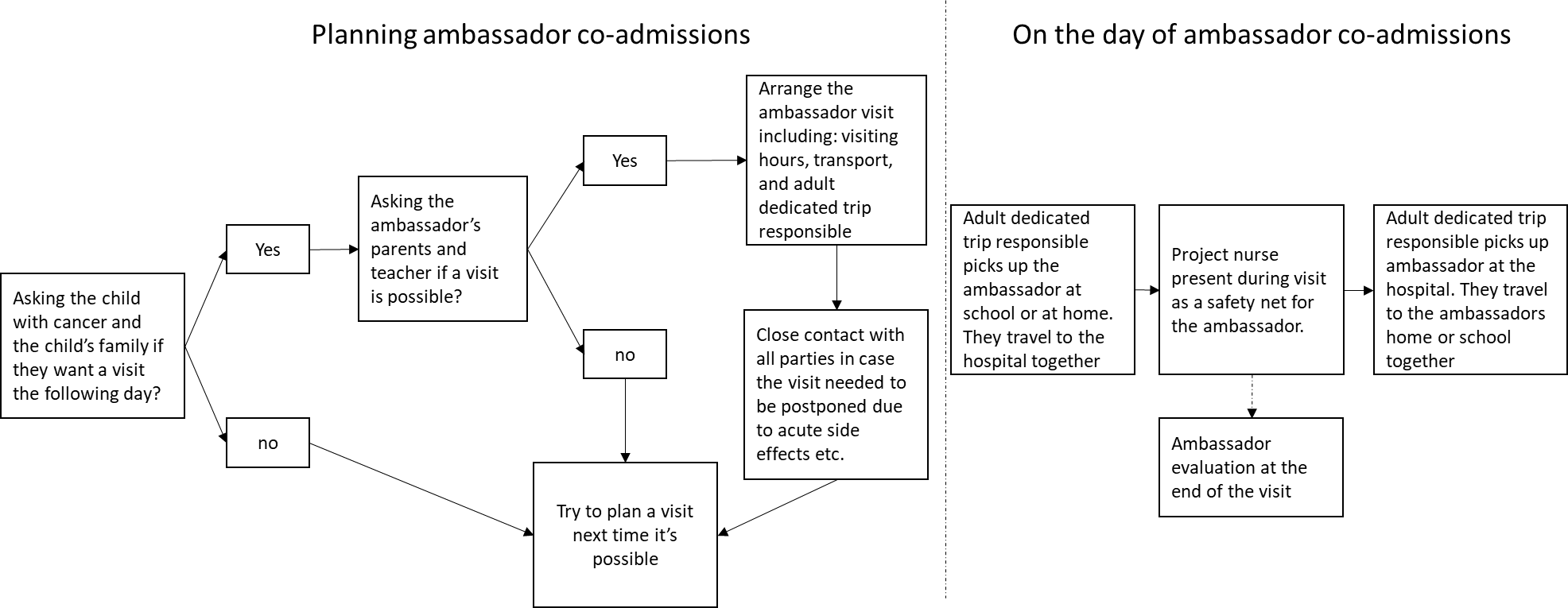

Supplement: Supplementary file 1 — Additional file 1. Flow chart of planning an ambassador co-admissions. [file 12916_2020_1634_MOESM1_ESM.docx]
