# Supplementary material for: Effects of a physical activity program from diagnosis on cardiorespiratory fitness in children with cancer: a national non-randomized controlled trial
Source: BMC Med. 2020 Jul 6;18:175. doi: 10.1186/s12916-020-01634-6 (PMC7336676; doi:10.1186/s12916-020-01634-6)
Supplement: Supplementary file 2 — Additional file 2. Exercise Library of which games were developed around: Overview of exercises primarily used in the RESPECT activity program. The primary focus was on Cardiorespiratory fitness, muscle strength, and stability/balance. [file 12916_2020_1634_MOESM2_ESM.docx]

**Additional file 2** Exercise Library of which games were developed around: Overview of exercises primarily used in the RESPECT activity program. The primary focus was on Cardiorespiratory fitness, muscle strength, and stability/balance.

| **Cardiorespiratory fitness exercises:** |
| --- |
| Steps |
| Bicycle ergometer |
| Treadmill |
| Cross trainer |
| Running / marching on the spot |
| Walks |
| Walking in intervals |
| High knee running |
| Heel kick running |
| Stairways |
| **Strength exercises** |
| **Lower-extremity exercises:** |
| Up on toes / walk on toes |
| Up on heels / walk on heels |
| Squat (air, front, Bulgarian) |
| Wall sit |
| Deadlift (both legs, single leg) |
| Thrusters |
| Lunges |
| Side step / shuffle |
| Side step / shuffle crossover |
| Skip |
| Walking backwards |
| Jumps |
| Jumping jacks |
| Frog jumps |
| Knees to elbows |
| Crawl (bear, crab) |
| Making turns |
|  |
| **Upper-extremity exercises:** |
| Push-ups |
| Fly |
| Rowing |
| Shoulder press |
| Flyers |
| Laterals |
| Dips |
| Dumbbell press |
| Pull-down |
| Biceps curl |
| Hammer curl |
| Push-down |
|  |
| **Core and pelvis exercises:** |
| Hip thruster |
| Plank |
| Back extensions |
| Abdominal crunch / sit-up |
| Leg lifts |
| Bicycle legs |
|  |
| **Stability/balance and cardiorespiratory fitness** |
| **Balance, stability and neuromuscular exercises:** |
| Stand on one leg |
| Walk in a straight line |
| Agility course |
| Balance on an unstable base (e.g. foam pad) |
| Balance board |
| ‘Throwing’ balls from unstable surfaces |
| Sitting and kneeling balance exercises on Swiss exercise ball (eyes open and closed) |
