# Supplementary material for: Effects of a physical activity program from diagnosis on cardiorespiratory fitness in children with cancer: a national non-randomized controlled trial
Source: BMC Med. 2020 Jul 6;18:175. doi: 10.1186/s12916-020-01634-6 (PMC7336676; doi:10.1186/s12916-020-01634-6)
Supplement: Supplementary file 3 — Additional file 3. Reasons why tests were not completed. Not safe to perform test was based on the treating physician’s evaluation of the safety of performing the tests. Unable to perform the test was based on an assessment of the child’s general well-being and physical capacity prior to testing. Not motivated if no apparent physical reason for not performing the test. Logistical reasons include concurrent medical procedures that prevented testing and receiving treatment abroad. Furthermore, the CPET test was not available at Odense University Hospital. Equipment issues include equipment failure and maintenance. Late inclusion includes children that were enrolled later in their treatment. [file 12916_2020_1634_MOESM3_ESM.docx]

**Additional file 3:** Reasons why tests were not completed

|  | **Not safe to perform test** | **Unable to perform test** | **Not motivated** | **Logistical reasons** | **Equipment issues** | **Late inclusion** |
| --- | --- | --- | --- | --- | --- | --- |
| **Cardiopulmonary exercise test (CPET)** |  | | | | | |
| **Intervention Group** |  |  |  |  |  |  |
| Baseline | 24 (20%) | 42 (36%) | 6 (5%) | 5 (4%) | 4 (3%) | 1 (1%) |
| Three Months | 20 (17%) | 50 (42%) | 12 (10%) | 7 (6%) | 0 (0%) | 1 (1%) |
| Six Months | 19 (16%) | 50 (42%) | 13 (11%) | 4 (3%) | 0 (0%) | 0 (0%) |
| **Control group** |  |  |  |  |  |  |
| Baseline | 3 (6%) | 14 (28%) | 1 (2%) | 5 (10%) | 0 (0%) | 14 (28%) |
| Three Months | 6 (12%) | 13 (26%) | 0 (0%) | 17 (34%) | 0 (0%) | 1 (2%) |
| Six Months | 9 (18%) | 11 (22%) | 2 (4%) | 16 (32%) | 0 (0%) | 0 (0%) |
| **Sit-To-Stand** | | | | | | |
| **Intervention Group** |  |  |  |  |  |  |
| Baseline | 10 (8%) | 13 (11%) | 4(3%) | 2 (2%) | 0 (0%) | 1 (1%) |
| Three Months | 11 (9%) | 18 (15%) | 11 (9%) | 5 (4%) | 0 (0%) | 1 (1%) |
| Six Months | 13 (11%) | 17 (14%) | 8 (7%) | 1 (1%) | 0 (0%) | 0 (0%) |
| **Control Group** |  |  |  |  |  |  |
| Baseline | 3 (6%) | 6 (12%) | 1 (2%) | 0 (0%) | 0 (0%) | 14 (28%) |
| Three Months | 6 (12%) | 2 (4%) | 0 (0%) | 3 (6%) | 0 (0%) | 1 (2%) |
| Six Months | 6 (12%) | 6 (12%) | 3 (6%) | 6 (12%) | 0 (0%) | 0 (0%) |
| **Timed-Up-and-Go** | | | | | | |
| **Intervention Group** |  |  |  |  |  |  |
| Baseline | 10 (8%) | 15 (13%) | 7 (6%) | 2 (2%) | 0 (0%) | 1 (1%) |
| Three Months | 11 (9%) | 19 (16%) | 10 (8%) | 5 (4%) | 0 (0%) | 1 (1%) |
| Six Months | 13 (11%) | 17 (14%) | 7 (6%) | 1 (1%) | 0 (0%) | 0 (0%) |
| **Control Group** |  |  |  |  |  |  |
| Baseline | 3 (6%) | 5 (10%) | 1 (2%) | 0 (0%) | 0 (0%) | 14 (28%) |
| Three Months | 5 (10%) | 2 (4%) | 0 (0%) | 3 (6%) | 0 (0%) | 1 (2%) |
| Six Months | 5 (10%) | 7 (14%) | 4 (8%) | 6 (12%) | 0 (0%) | 0 (0%) |
| **Flamingo balance** | | | | | | |
| **Intervention Group** |  |  |  |  |  |  |
| Baseline | 5 (4%) | 13 (11%) | 4 (3%) | 2 (2%) | 0 (0%) | 1 (1%) |
| Three Months | 6 (5%) | 14 (12%) | 8 (7%) | 5 (4%) | 0 (0%) | 1 (1%) |
| Six Months | 9 (8%) | 18 (15%) | 5 (4%) | 1 (1%) | 0 (0%) | 0 (0%) |
| **Control Group** |  |  |  |  |  |  |
| Baseline | 3 (6%) | 5 (10%) | 1 (2%) | 0 (0%) | 0 (0%) | 14 (28%) |
| Three Months | 5 (10%) | 2 (4%) | 1 (2%) | 3 (6%) | 0 (0%) | 1 (2%) |
| Six Months | 5 (10%) | 7 (14%) | 4 (8%) | 6 (12%) | 0 (0%) | 0 (0%) |
| **Handgrip strength (Right)** | | | | | | |
| **Intervention Group** |  |  |  |  |  |  |
| Baseline | 5 (4%) | 7 (6%) | 2 (2%) | 2 (2%) | 0 (0%) | 1 (1%) |
| Three Months | 6 (5%) | 11 (9%) | 7 (6%) | 5 (4%) | 0 (0%) | 1 (1%) |
| Six Months | 7 (6%) | 17 (14%) | 4 (3%) | 1 (1%) | 0 (0%) | 0 (0%) |
| **Control Group** |  |  |  |  |  |  |
| Baseline | 2 (4%) | 5 (10%) | 1 (2%) | 0 (0%) | 0 (0%) | 14 (28%) |
| Three Months | 4 (8%) | 2 (4%) | 0 (0%) | 3 (6%) | 0 (0%) | 1 (2%) |
| Six Months | 4 (8%) | 6 (12%) | 3 (6%) | 6 (12%) | 0 (0%) | 0 (0%) |
| **Handgrip strength (Left)** | | | | | | |
| **Intervention Group** |  |  |  |  |  |  |
| Baseline | 3 (3%) | 7 (6%) | 3 (3%) | 2 (2%) | 0 (0%) | 1 (1%) |
| Three Months | 6 (5%) | 11 (9%) | 7 (6%) | 5 (4%) | 0 (0%) | 1 (1%) |
| Six Months | 7 (6%) | 17 (14%) | 4 (3%) | 1 (1%) | 0 (0%) | 0 (0%) |
| **Control Group** |  |  |  |  |  |  |
| Baseline | 2 (4%) | 5 (10%) | 1 (2%) | 0 (0%) | 0 (0%) | 14 (28%) |
| Three Months | 4 (8%) | 2 (4%) | 0 (0%) | 3 (6%) | 0 (0%) | 1 (2%) |
| Six Months | 4 (8%) | 6 (12%) | 3 (6%) | 6 (12%) | 0 (0%) | 0 (0%) |

**Not safe to perform test** was based on the treating physician’s evaluation of the safety of performing the tests. **Unable to perform the test** was based on an assessment of the child’s general well-being and physical capacity prior to testing. **Not motivated** if no apparent physical reason for not performing the test. **Logistical reasons** include concurrent medical procedures that prevented testing and receiving treatment abroad. Furthermore, the CPET test was not available at Odense University Hospital. **Equipment issues** include equipment failure and maintenance. **Late inclusion** includes children that were enrolled later in their treatment.
