# Supplementary material for: Effects of a physical activity program from diagnosis on cardiorespiratory fitness in children with cancer: a national non-randomized controlled trial
Source: BMC Med. 2020 Jul 6;18:175. doi: 10.1186/s12916-020-01634-6 (PMC7336676; doi:10.1186/s12916-020-01634-6)
Supplement: Supplementary file 4 — Additional file 4. Effects of the RESPECT activity program: Results from the linear mixed model. The first mentioned covariable serves as the reference variable in the linear mixed model. P < 0.017 was considered significant. INT = intervention group, CON = Control group. Oncologic consists of oncological diseases (extracranial solid tumors and tumors located in central nervous system). Hematologic consists of hematological diseases (leukemia, lymphoma, Langerhans cell histiocytosis and myelodysplastic syndrome). [file 12916_2020_1634_MOESM4_ESM.docx]

Additional file 4: Effects of the RESPECT activity program: Results from the linear mixed model.

|  | **VO_2peak_ (mL/kg/min)** | | | **Watt max (W)** | | |
| --- | --- | --- | --- | --- | --- | --- |
|  | Estimate | 95% CI | p-value | Estimate | 95% CI | p-value |
| Age  change per year | -0.17 | -0.62 to 0.28 | 0.4569 | 9.70 | 7.29 to 12.10 | **<0.0001** |
| Sex  (Females vs Males) | -2.95 | -5.77 to -0.12 | 0.041 | -16.94 | -31.94 to -1.95 | **0.0273** |
| Disease  (Oncologic vs Hematologic) | 4.71 | 1.85 to 7.60 | 0.0016 | 8.07 | -7.12 to 23.27 | 0.2931 |
| Effect of time per week  CON | -0.19 | -0.35 to -0.03 | 0.0183 | -1.03 | -1.88 to -0.18 | 0.0185 |
| Effect of time per week  INT | 0.06 | -0.02 to 0.15 | 0.1415 | 0.44 | -0.04 to 0.91 | 0.0694 |
| Effect of time at Baseline  (INT vs CON) | -2.11 | -6.10 to 1.81 | 0.2830 | -21.46 | -43.05 to -0.64 | 0.0438 |
| Effect of time at 3-months  (INT vs CON) | 1.16 | -1.79 to 4.16 | 0.4316 | -2.62 | -18.48 to 13.15 | 0.7382 |
| Effect of time at 6-months  (INT vs CON) | 4.43 | 0.92 to 8.11 | 0.0146 | 16.23 | -2.71 to 35.72 | 0.0912 |
| Interaction grp * time per week | 0.25 | 0.07 to 0.43 | 0.0062 | 0.21 | 0.07 to 0.35 | **0.0038** |
| **Timed-Up-and-Go (%)** | | | **Sit-To-Stand** | | | |
|  | Estimate | 95% CI | p-value | Estimate | 95% CI | p-value |
| Age  change per year | -2.2 | -3.2 to -1.1 | **0.0001** | 0.04 | -0.26 to 0.35 | 0.7709 |
| Sex  (Females vs Males) | 3.2 | -2.2 to 10.9 | 0.3725 | 1.43 | -0.58 to 3.44 | 0.1614 |
| Disease  (Oncologic vs Hematologic) | -8.7 | -16.0 to 0.6 | **0.0189** | 3.08 | 1.03 to 5.13 | **0.0035** |
| Effect of time per week  CON | 0.22 | -0.2 to 0.6.5 | 0.3038 | -0.04 | -0.15 to 0.07 | 0.4960 |
| Effect of time per week  INT | 0.02 | -0.2 to 0.2 | 0.8537 | 0.004 | -0.06 to 0.06 | 0.8919 |
| Effect of time at Baseline  (INT vs CON) | -29.3 | -40.4 to -19.3 | **<0.0001** | 6.62 | 3.86 to 9.62 | **<0.0001** |
| Effect of time at 3-months  (INT vs CON) | -31.9 | -40.2 to -24.8 | **<0.0001** | 7.19 | 5.31 to 9.65 | **<0.0001** |
| Effect of time at 6-months  (INT vs CON) | -34.5 | -44.6 to -25.7 | **<0.0001** | 7.75 | 5.31 to 10.46 | **<0.0001** |
| Interaction grp * time | 0.02 | -0.09 to 0.04 | 0.4129 | 0.04 | -0.08 to 017 | 0.5057 |
| **Right Handgrip strength (kg)** | | | **Left Handgrip strength (kg)** | | | |
|  | Estimate | 95% CI | p-value | Estimate | 95% CI | p-value |
| Age  change per year | 0.21 | -0.36 to 0.79 | 0.4691 | 2.43 | 2.13 to 2.72 | <0.0001 |
| Sex  (Females vs Males) | -4.11 | -7.87 to -0.35 | 0.0323 | -3.09 | -5.03 to -1.17 | 0.0019 |
| Disease  (Oncologic vs Hematologic) | 3.14 | -0.65 to 6.94 | 0.1038 | 2.68 | 0.74 to 4.62 | 0.0071 |
| Effect of time per week  CON | -0.10 | -0.35 to 0.14 | 0.4085 | -0.11 | -0.19 to -0.03 | 0.0062 |
| Effect of time per week  INT | -0.11 | -0.23 to 0.14 | 0.0711 | -0.04 | -0.08 to -0.001 | 0.0466 |
| Effect of time at Baseline  (INT vs CON) | 4.08 | -1.66 to 6.65 | 0.3418 | 3.85 | 1.42 to 6.40 | 0.0032 |
| Effect of time at 3-months (INT vs CON) | 2.72 | -1.43 to 6.97 | 0.1959 | 4.62 | 2.60 to 6.81 | <0.0001 |
| Effect of time at 6-months (INT vs CON) | 2.59 | -2.46 to 7.72 | 0.3087 | 5.53 | 3.31 to 7.95 | <0.0001 |
| Interaction grp * time | -0.010 | -0.29 to 0.27 | 0.9438 | 0.01 | -0.002 to 0.02 | 0.1204 |

The first mentioned covariable serves as the reference variable in the linear mixed model. P<0.017 was considered significant. INT= intervention group, CON= Control group. Oncologic consists of oncological diseases (extracranial solid tumors and tumors located in central nervous system). Hematologic consists of hematological diseases (leukemia, lymphoma, Langerhans cell histiocytosis and myelodysplastic syndrome)
